# Compensability of Enhanced Cytoplasmic Droplet Rates in Boar Semen: Insights of a Retrospective Field Study

**DOI:** 10.3390/ani12202892

**Published:** 2022-10-21

**Authors:** Martin Schulze, Dagmar Waberski

**Affiliations:** 1 Institute for Reproduction of Farm Animals Schönow, Bernauer Allee 10, D-16321 Bernau, Germany; 2 Unit for Reproductive Medicine of Clinics, Clinic for Pigs and Small Ruminants, University of Veterinary Medicine Hannover, Bünteweg 15, D-30559 Hannover, Germany

**Keywords:** artificial insemination, boar semen, cytoplasmic droplets, cold resistance, heat resistance, sperm morphology

## Abstract

**Simple Summary:**

Cytoplasmic droplets (CD) represent a common sperm abnormality in boar. Their negative effect on fertility might be compensated by high numbers of morphological and functional intact spermatozoa in the insemination dose. To prove this hypothesis, a retrospective analysis of fertility data from 1497 inseminations obtained with 260 extended ejaculates of 130 boar was performed. CD rates > 11% provided a greater risk for reduced litter sizes. However, average litter sizes in the semen group with high CD rate (>15%) did not differ from groups with medium (10–15%) or low (<10%) CD rates. The farrowing rate was even slightly higher in the high CD group. This was accompanied by a higher tolerance of sperm to heat and cold stress compared to semen groups with lower CD rates. Other functional sperm parameters did not differ between the three CD groups. In conclusion, an increased rate of CD in extended boar semen portions can be compensated by a high percentage of stress-tolerant sperm so that fertility is not affected.

**Abstract:**

Retained cytoplasmic droplets (CD) provide the most abundant sperm abnormality in boar and reduce fertility. It is still unclear as to whether high CD rates in semen portions are compensable. The aim was to explore the impact of CD in relation to quantitative and qualitative sperm traits on fertility performance of sows. Retrospective data analysis of 1497 inseminations was performed. Ejaculates (*n* = 260) were assigned to three groups with low (<10%), medium (≥10% to <15%), and high (≥15%) CD rates. Average sperm numbers were lowest in the high CD group (2.08 × 10^9^/mL). Membrane integrity and mitochondrial activity did not differ between the groups. Breakpoint analysis indicated a shift towards lower litter sizes when the CD rate exceeded 11%. Group comparisons revealed no difference in litter size (*p* = 0.205), together with a slightly higher farrowing rate in the high CD group (*p* < 0.001), which coincided with higher resistance against temperature stress in the stored semen samples (*p* < 0.001) and a higher sperm motility (*p* < 0.001). In conclusion, an increased prevalence of CD in boar semen is compensable by high tolerance against temperature stress, whereas sperm numbers per dose are less relevant.

## 1. Introduction

Spermatozoa with retained cytoplasmic droplets present the most frequent morphological abnormality in boar semen. Failure to shed cytoplasmic droplets from epididymal sperm during ejaculation is regarded as a sign of sperm immaturity [1], or alternatively could be associated with a disturbance in spermiogenesis [2]. Seasonal increases in CD in boar semen affect economic production of semen doses and may impede availability of the requested genetics for sow breeding. Regardless of the etiology, enhanced prevalence of spermatozoa with CD reduces pregnancy rates, farrowing rates, and litter sizes [3,4,5,6]. For this reason, many breeding organizations have set upper limits between 15% and 30% for sperm with CD in insemination portions [7]. The definition of such limits is under debate, as other breeding organizations have not defined specific thresholds for CD-rates. The controversy is prompted by recent studies indicating that an enhanced prevalence of CD could be compensated by higher numbers of morphologically and functionally intact spermatozoa in the semen dose [8,9]. Whether loss of fertility in vivo can thus be avoided in semen doses with elevated presence of CD remains to be shown. 

Compensation of sperm defects by an increase in total sperm numbers in the semen dose is well described for abnormalities which hinder the sperm’s access to the oocytes [10,11], for example sperm with bent tails. The observation that semen samples with high incidence of CD have a lower binding capacity to oviductal epithelium in vitro [12,13] and that sperm with CD show reduced linear sperm movement [9] indicate that, similar to bent tails, these sperm are excluded from interaction with the oocytes in favor of morphologically intact, competent sperm. Proof of the hypothesis that an increased incidence of CD is a compensable sperm defect with in vivo fertility data is difficult to achieve because semen effects on field fertility are small due to predominant effects related to the farm management and sows [11,14]. Consequently, large data sets of fertility records or well-controlled insemination trials with documented in vitro sperm quality data and sperm numbers per semen dose are required to allow reliable conclusions on fertility outcome [11,15]. 

A recent well-standardized field insemination study published by Schulze et al. [16] offered the chance for a retrospective analysis of a relatively large data set with a special focus on CD. In the cited field trial, detailed sperm characteristics, including resistance to temperature stress, and fertility data of single sire inseminations were recorded from 260 extended ejaculates of 130 young Piétrain boar. Beyond this background, the aim of the present study was to elucidate the relevance of CD for fertility performance by a retrospective analysis of field insemination data collected in 1497 sows. Our hypothesis was that semen samples with a high incidence of CD (>15%) can achieve high fertility results due to compensation for this defect. Breakpoint analysis was performed to determine whether and at which starting point the CD rate shifts towards smaller farrowing rates or litter sizes. Overall, the objective was to examine the effect of elevated incidence of CD on fertility performance in sow herds and to update recommendations for the utilization of semen samples with this sperm abnormality in artificial insemination.

## 2. Materials and Methods

### 2.1. Boar and Semen Samples

Semen analysis data and fertility records previously collected by Schulze et al. [16] were re-analyzed. Ethical approval for the original study [16] was obtained from the animal welfare committee of the Institute for Reproduction of Farm Animals Schönow (IFN -2019-V-05). Data comprised of semen extended in Beltsville Thawing Solution (BTS); (DiluPorc™, Sinus, Heidelberg, Germany) from the respective fourth and fifth ejaculate of 130 clinically healthy young Piétrain boar (aged eight to nine months) from one boar stud. Only ejaculates with a minimum sperm motility of 70% were processed. Semen dose volumes were 87.6 ± 1.5 mL and contained 2.25 ± 0.51 × 10^9^ sperm per tube. Semen doses were transported at 16–18 °C in insulated, temperature-controlled boxes to the IFN laboratory and to three sow farms.

### 2.2. Semen Quality Records

Evaluation of semen samples was performed at the reference laboratory of the IFN as described in detail by Schulze et al. [16]. In brief, sperm concentration in the semen doses was determined with the NucleoCounter SP-100™, and the volume of semen doses was measured to calculate the total sperm number per dose. Sperm morphology was assessed in 200 sperm of formaline-fixed samples using phase-contrast microscopy at 800× magnification. Sperm motility (total) was evaluated in a minimum of 400 sperm using the computer-assisted sperm analysis (CASA) system AndroVision^®^ (Version 1.1, Minitüb, Tiefenbach, Germany). Semen samples were analyzed in a pre-warmed Leja chamber (Leja Products B.v., Nieuw-Vennep, The Netherlands) in four to five frames (100× magnification) at a rate of 30 pictures per 0.5 s. Mitochondrial activity, acrosomal status, and membrane integrity were evaluated using a Cytoflex S flow cytometer (Beckman Coulter GmbH, Krefeld, Germany). Viable sperm with active mitochondria were determined by propidium iodide (PI) -negative and rhodamine 123-positive stained cells. The percentage of viable sperm with intact acrosomal membranes were determined by PI-negative and PNA- and PSA-negative cells. 

### 2.3. Heat and Cold Resistance of Semen Samples

Temperature resistance of semen samples were determined as described in detail by Schulze et al. [16]. The cold resistance test (CRT) was performed in which sperm motility was determined in the extended semen samples after storage at 6 °C for three days. In the heat resistance test (HRT), sperm motility was assessed in the extended semen samples after storage at 17 °C for seven days and subsequent incubation in a water bath at 38 °C for 5 h. 

### 2.4. Fertility Records

Fertility data were evaluated from routine insemination of crossbred sows from three farms as described previously [16]. On all farms, estrus was detected twice daily in the presence of teaser boar, and sows were first inseminated 24 h after the first positive back pressure test. If sows were still in estrus 12 h later, a second insemination was performed. Gilts and sows inseminated with semen from more than one boar per cycle were excluded from analysis. The final data set comprised 1497 insemination records. 

### 2.5. Statistical Analysis

Data analysis was performed using SigmaPlot 14.0 (Systat Software Inc., Point Richmond, CA, USA). Descriptive data were expressed as mean, standard deviation (SD), minimum, median, and maximum. The data set was divided into three groups according to the rate of CD: Group A: low, <10% CD (*n* = 1235 insemination records), Group B: medium, ≥10% to <15% CD (*n* = 125 insemination records), and Group C: high, ≥15% CD (*n* = 137 insemination records). Group C was defined as ‘high’ based on current recommendation for useable semen which set 15% CD as upper limit value [3,7]. Statistical analysis of fertility and spermatological data were performed by one-way analysis of variance on ranks. When ANOVA on ranks revealed a significant difference between the Groups (A–C), the values were compared using the Dunn’s multiple comparison test (post hoc) with Bonferroni correction. Differences among medians were considered to be statistically significant when *p* ≤ 0.05. To find possible breakpoints (T1, change points) for CD rate, predicting the fertilizing potential of AI boar, a two segmental non-linear regression analysis was carried out. The calculation of the segments was based on the Marquardt–Levenberg algorithm [17]. The following algorithm was used for the analysis with CD rate as independent variables (*t*) and number of total born piglets as dependent factors (*y*):

$$f=\{\begin{array}{c} y_{1}\times \left(T_{1}-t\right)+y_{2}\times \left(t-t_{1}\right))/\left(T_{1}-t_{1}\right)\\ y_{2}\times \left(t_{2}-t\right)+y_{3}\times \left(t-T_{1}\right))/\left(t_{2}-T_{1}\right) \end{array}$$

$$t_{1}=min\left(t\right),t_{2}=max\left(t\right),f=if\left(t\leq T_{1},region1\left(t\right),region2\left(t\right)\right)$$

## 3. Results

### 3.1. Descriptive Statistics of Semen Related Factors

**Figure 1:**
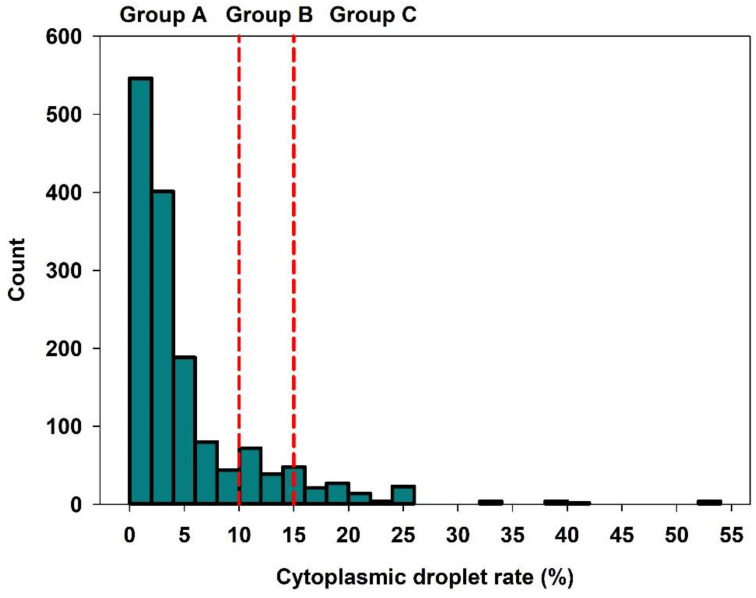
Frequency distribution of the cytoplasmic droplet rate within the study population (*n* = 260 ejaculates, two per boar; *n* = 1497 insemination records). Semen samples were assigned to three groups with different CD rates: A: low (<10%), B: medium (≥10%–<15%), and C: high (≥15%).

Semen quality characteristics, including heat and cold resistance, are reported in Table 1. Quantitative and qualitative semen attributes were in the expected range for semen used for insemination on breeding sow farms. Motility after heat and cold stress in stored semen samples decreased to 52% on average compared to non-stressed controls at day 3 (mean: 82%). The frequency distribution of semen samples in the groups of low, moderate and high CD rate is shown in Figure 1. A total of 80.8% (*n* = 210 ejaculates) of the semen samples were assigned to Group A (low CD-rate), 8.8% (*n* = 23 ejaculates) to Group B (moderate CD rate), and 10.4% (*n* = 27 ejaculates) of the samples were present in Group C (high CD rate). The distribution of samples to the three groups was in a similar range within the three farms: Group A: 81.8–90.6%, Group B: 2.0–9.0%, Group C: 7.6–9.3% of the semen samples. 

### 3.2. Descriptive Statistics of Sow Related Factors

The sow-related data set of 1497 insemination records is shown in Table 2. Data were extracted from the sow management software “db.Planer” (version 20/11, BHZP GmbH, Dahlenburg-Ellringen, Germany). The values of fertility descriptors were in the expected range of routinely performed inseminations on the farms and did not differ between the three CD groups (Table 3a).

### 3.3. Relation between the CD Rate and Fertility

The comparison of the groups low (A), moderate (B), and high (C) CD rates revealed statistically significant differences in farrowing rate in favor of the group with high prevalence of CD (A vs. B: *p* = 1.000, A vs. C: *p* = 0.003, B vs. C: *p* = 0.006). All other fertility indicators did not differ (*p* > 0.05) between the CD groups (Table 3b). Within Group C, insemination (*n* = 28) using semen portions with >25% CD (processed from seven ejaculates of different boar) resulted in a 92.9% farrowing rate and 14.6 ± 4.1 piglets (total). Table 4 shows the group comparisons for semen-related factors. Here, significant differences (*p* ≤ 0.05) were found in the following parameters: total sperm number per dose, total sperm motility, sperm morphology, and heat and cold resistance. Semen samples in Group C (high CD rate) had a higher total sperm motility compared to both other groups (A vs. B: *p* = 0.217, A vs. C: *p* < 0.001, B vs. C: *p* = 0.009). The total sperm number per dose was significantly lower in Group C samples compared with Group A (A vs. B: *p* = 0.297, A vs. C: *p* < 0.001, B vs. C: *p* = 0.026; Table 4a). Furthermore, the cold resistance of the samples in Group C was significantly higher (cold resistance: A vs. B: *p* = 1.000, A vs. C: *p* < 0.001, B vs. C: *p* = 0.008). Comparison of the heat resistance showed better results for Groups B and C compared to A (A vs. B: *p* = 0.003, A vs. C: *p* < 0.001, B vs. C: *p* = 1.000). There were no differences in mitochondrial activity and membrane integrity between the three groups (Table 4b).

**Figure 2:**
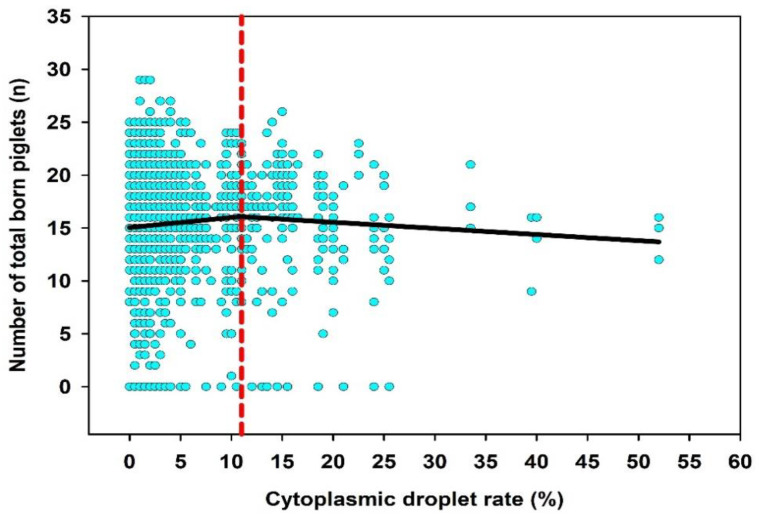
Two segmental non-linear regression analysis for the parameter cytoplasmic droplet rate (*n* = 260 ejaculates, two per boar; *n* = 1497 inseminations records).

Segmental non-linear regression analysis was performed to determine potential breakpoints of the CD rate which could be predictive for the fertilizing performance of the boar. For this, data sets were divided step by step into two to five segments. Only results of the two segmental non-linear regression analyses were presented, because the division into more than two segments did not yield any further information. It could be shown that a CD rate > 11% was classified as downshifting the number of total born piglets (Figure 2). For the fertility indicator farrowing rate, no meaningful breakpoints were found in the data distribution regarding the incidence of CD in the semen samples. Group comparisons between ≤11% CD (*n* = 1299) and >11% CD (*n* = 198) did not show significant differences in litter sizes and farrowing rates (*p* > 0.05).

## 4. Discussion

This retrospective analysis of semen characteristics and fertility data demonstrates that an enhanced prevalence of CD is not necessarily associated with reduced fertility, probably due to compensation resulting from a higher stress tolerance of the preserved semen sample. Conversely, farrowing rates were even slightly higher in the group with the highest prevalence of CD, even though the fertility levels on all three farms were high. Nonetheless, in accordance with earlier reports [3], CD rates above 11% were identified as a shift point towards smaller litter sizes, which, however, remained statistically inapparent in the average values for total born piglets. 

Retained CD impair sperm function by enhanced peroxidation of membrane lipids [18] through production of reactive oxygen species [19], increased membrane destabilization during in vitro storage [20] and impaired in vitro capacitation [19,20]. In the present study, compensation of impaired sperm function was not achieved by higher sperm number in the semen dose. In fact, just the opposite, total sperm numbers being even lower in the CD-rich semen group, thus pointing to other compensatory mechanism. An interesting finding is that motility after temperature stress in preserved semen samples was high in the CD-rich semen group. At this point, a causative relation between the incidence of CD and sperm’s resistance to heat or cold stress cannot be established, but obviously higher CD rates do not compromise the stress tolerance of the semen sample. 

Previous analysis of field data revealed that temperature stress tests have predictive value for fertility [16]. In this case, it can be suggested that a high resistance of sperm to temperature stress could compensate for an enhanced incidence of morphological abnormal sperm in the preserved semen sample. Compensation would thus be possible not only on a quantitative basis through enhanced sperm numbers in the semen dose [11,21], but alternatively or in addition to a qualitative basis through a high stress tolerance. Noteworthy, other characteristics of sperm function (mitochondrial activity) and viability (membrane integrity) were on a similarly high level in the three CD groups, which might be a necessity to allow compensation. 

As the present, results indicate that temperature-based stress tests might become a valuable tool for testing for semen usability in the case of above moderate threshold values for distinct morphological sperm abnormalities in otherwise functionally competent semen samples. Before implementing these tests in AI laboratories, however, the boar-specific consistency of stress resistance in preserved semen samples should be shown [22]. Previous data indicated a clear boar specificity to withstand stress resulting from semen cryopreservation and sex-sorting [23]. Further research should evaluate whether this also applies for temperature stress tests. Heat and cold resistance of semen samples are only moderately related to each other (*r_s_* = 0.40, *p* < 0.001, *n* = 260) and might cover different sperm attributes with relevance for fertility [16]. To this point, both test types should be included in larger scale studies. 

Data of the present study are based on young Piétrain boar aged between eight and nine months, which just have reached sexual maturity and qualified as AI boar. In contrast to the quantitative traits, semen volume and sperm concentration, the sperm morphology, particularly the rate of cytoplasmic droplets is relatively stable in boar aged between eight and 17 months [24]. Thus, if inter-boar variance is shown, the stress test could be implemented already in the selection procedure for young boar. For this, breed difference should be taken into account, especially considering semen of Duroc boar which have a relatively high CD incidence [24,25]. 

## 5. Conclusions

In semen doses containing ≥2 × 10^9^ sperm, an increased prevalence of CD (>15%) in boar semen is compensable by high tolerance against temperature stress in samples with high motility. Functional sperm attributes based on temperature tolerance could represent a valuable, easily utilized tool to assess the compensability of increased CD rates, especially when boar consistency is proven. Limit values for CD before fertility becomes affected were not identified in the present retrospective analysis and will depend on the sperm number per dose. Nonetheless, specific threshold values for CD in minimum standards for sperm morphology as defined by several breeding organizations [7] seem dispensable.

## Figures and Tables

**Table 1:** Descriptive statistics of semen-related factors (*n* = 260 ejaculates, two per boar).

Sperm Quality Characteristics	Day ^1^	Mean	SD	Min	Median	Max
Semen volume, mL	0	214.9	72.7	20.0	205.0	408.0
Sperm concentration, 10^9^/mL	0	0.313	0.122	0.119	0.284	0.874
Sperm output, 10^9^	0	58.6	18.4	9.8	57.4	119.9
Dose volume, mL	1	87.6	1.5	83.4	87.5	92.5
Total sperm number per dose, 10^9^	1	2.24	0.48	1.15	2.24	5.06
Mitochondrial active sperm, %	2	76.2	6.6	53.9	76.8	88.2
Membrane intact sperm, %	2	78.0	6.5	55.9	78.5	89.7
Morphologically normal sperm, %	3	79.4	13.1	30.5	81.5	99.0
Cytoplasmic droplet rate, %	3	5.0	6.4	0	2.5	52.0
Total sperm motility, %	3	81.7	15.3	15.5	87.7	96.9
Cold-resistant sperm, %	3	51.9	16.4	18.8	50.6	91.9
Heat-resistant sperm, %	7	52.1	23.2	8.2	58.6	84.9

^1^ Day 0: native semen; D 1 to 7: storage day of semen extended in Beltsville Thawing Solution.SD = standard deviation, Min = minimum, Max = maximum.

**Table 2:** Descriptive statistics of sow-related factors (*n* = 1497 inseminations records).

Parameter	Mean	SD	Min	Median	Max
Parity	4.2	2.4	1.0	4.0	13.0
Weaning-to-estrus interval, d	6.0	5.8	2.0	4.0	54.0
Gestation length, d	115.7	1.6	79.0	116.0	120.0
Farrowing rate, %	94.9	7.1	50.0	100.0	100.0
Lactation period, d	26.0	4.2	0	27.0	48.0
Total number of piglets born, n	15.4	5.3	0	16.0	29.0
Number of live born piglets, n	14.7	3.7	1.0	15.0	27.0
Number of stillborn piglets, n	1.5	2.0	0	1.0	18.0
Number of piglets weaned per litter, n	11.3	2.6	0	12.0	34.0
Farrowing interval, d	149.1	6.6	127.0	147.0	204.0

SD = standard deviation, Min = minimum, Max = maximum.

**Table 3:** Sow-related factors (**a**) and fertility data (**b**) regarding the three different levels of cytoplasmic droplet (CD) rates in the semen used for insemination: A: low (<10%), B: moderate (≥10 and <15%) and C: high (≥15%). Values are shown as median (interquartile range). Significant differences between the groups are marked by different superscript index letters (a, b).

(**a**)					
**CD Group**	**Sows (*n*)**	**Parity**	**Lactation Period (d)**	**Weaning-to-Estrus Interval (d)**	**Gestation Length (d)**
**A**	1235	4 (2–6)	27 (25–27) ^a^	4 (4–5) ^a^	116 (115–117)
**B**	125	4 (2–6)	27 (26–27) ^a^	4 (4–5) ^a^	116 (115–116)
**C**	137	4 (2–6)	27 (26–28) ^a^	5 (4–5) ^a^	115 (115–116)
***p*** **-value**		0.114	0.039	0.036	0.117
(**b**)					
**CD Group**	**Sows (*n*)**	**Farrowing Rate (%)**	**Total Number of Piglets Born**	**Number of Live Born Piglets**	**Number of StillbornPiglets**
**A**	1235	96.0 (90.9–100) ^a^	16 (13–18)	15 (13–17)	1 (0–2) ^a^
**B**	125	94.4 (88.9–100) ^a^	17 (14–19)	15.5 (13–17)	1 (0–3) ^a^
**C**	137	100(93.8–100) ^b^	16 (14–19)	15 (13–17)	1 (0–2) ^a^
***p*-value**		<0.001	0.205	0.806	0.044

**Table 4:** Standard sperm traits (**a**) and functional sperm traits (**b**) regarding three different levels of cytoplasmic droplet (CD) rates in the semen used for insemination: A: low (<10%), B: moderate (≥10% and <15%) and C: high (≥15%). Values are shown as median (interquartile range). Significant differences between the groups are marked by different superscript index letters (a, b).

(**a**)					
**CD** **Group**	**Sows (*n*)**	**Total Sperm Number per Dose (10^9^)**	**Total Sperm Motility (%)**	**Morphologically Normal Sperm (%)**	
**A**	1235	2.28 (1.95–2.56) ^a^	87.3 (80.5–91.1) ^a^	85.5 (74.5–91.5) ^a^	
**B**	125	2.20 (2.00–2.35) ^a,b^	88.9 (82.1–92.2) ^a^	70.5 (61.5–75.3) ^b^	
**C**	137	2.08 (1.77–2.24) ^b^	91.7 (84.3–93.4) ^b^	63.5 (60.3–70.5) ^b^	
***p*** **-value**		<0.001	<0.001	0.002	
(**b**)					
**CD** **Group**	**Sows (*n*)**	**Mitochondrial Active Sperm (%)**	**Membrane Intact Sperm (%)**	**Heat-Resistant Sperm (%)**	**Cold-Resistant Sperm (%)**
**A**	1235	77.0 (71.8–81.9) ^a^	78.5 (74.1–83.0)	56.7 (31.0–72.4) ^a^	48.7 (38.6–66.1) ^a^
**B**	125	76.7 (73.6–79.8) ^a^	79.9 (73.8–81.2)	62.5 (48.1–75.7) ^b^	52.0 (39.1–60.4) ^a^
**C**	137	75.1 (71.4–79.3) ^a^	76.9 (73.3–81.3)	60.8 (50.0–72.7) ^b^	60.5 (49.6–71.5) ^b^
***p*** **-value**		0.019	0.466	<0.001	<0.001

Heat resistance is defined as total sperm motility (%) in extended semen incubated for 5 h at 38 °C after storage for 7 days at 17 °C. Cold resistance is defined as total sperm motility (%) in extended semen stored for 3 days at 6 °C.

## Data Availability

The data presented in this study are available on request from the first author.
